# Dental Caries

**Published:** 1879-11

**Authors:** S. M. Prothro

**Affiliations:** Chatanooga, Tenn.


THE DENTAL REGISTER.

VOL. XXXIII.] NOVEMBER, 1879. NO. 11.

### DENTAL CARIES.

BY S. M. PROTHRO, OF CHATTANOOGA, TENN.

*Read Before the Tennessee Dental Association.*

Gentlemen: — Instead of delivering an address, as has been customary, in retiring from the position of presiding officer of our association, I felt that I might accomplish more good by the preparation of, and submitting for your consideration, a paper, on "Dental Caries," a subject of the most vital importance to every dentist who feels the least disposition to do good to his fellow man.

In the language of our gifted Thackston, the whole atmosphere of dental life, is warm with provocations to think and to act.

This subject, "Dental Caries," covers such a wide field, and embraces such a diversity of opinion, that it will be impossible, for me to do more than touch upon the principal points.

And at the outset, I must state frankly that I do not propose to offer much, if anything, that is new, or to surprise you with startling revelations, but simply to give you the benefit of my conclusions, after extensive reading, and critical investigation of this all important subject.

Caries of the teeth, is the most familiar expression of disease that engages the attention of the dentist, but its cause, progress and final result, have ever been enshrouded with uncertainty, and great diversity of opinion. When we sum up all the theories, after a century of research, aided by all the appliances of modern science, can we tell with absolute certainty the exact cause, or causes of decay of the human teeth?

Every cause has been assigned that could originate in the most fertile imagination.

One writer will tell us that it is acid, another declare that it is alkalies, one that it is local, another that is constitutional; one that it is mechanical, another that it is chemical, while others have come to the conclusion that animal or vegetable parasites and infusoria, being found in diseased teeth, through the disclosures of the microscope, must play some part in their disorganization and decay.

Before proceeding directly upon the subject under review we will give the constituents of the teeth, and the saliva.

According to Berzelius the teeth are composed of phosphate of lime, carbonate of lime, fluoride of lime, phosphate of magnesia, chloride of soda, gelatine and water, all of which salts, with the exception of the fluoride of lime are soluble in the acids found in the saliva.

Physiology teaches us that the saliva should have an alkaline reaction, in order to protect the teeth against acid taken as food, or arising from any other cause, but do we find it so; in the majority of cases it is acid. It is stated by some writer, that in one hundred cases, about seventy-five will be acid, twenty will be alkaline, and the remainder neutral.

The acids, which are incorporated in the saliva, are hydrochloric, nitric, sulphuric, carbonic, acetic, oxalic, uric, and lactic.

The alkalinity of the saliva is doubtless due to the presence of the salts, of lime and soda, in the form of phosphates.

There are other substances that may be detected in the saliva such as cholestrine, ptyaline, albumen, sulpho-cyanide of potash (which is a secretion of the sublingual gland) grape sugar, and even pus cells and blood corpuscles are discovered by the microscope.

We see then that the teeth are submerged in, and constantly exposed to agents, that it would seem are calculated to destroy them. But we will attempt to prove that something more is required than the decomposing effect of acids to complete the destruction of the human teeth.

Dental caries consists in a pathological process by which the tissues, are more or less softened, with gradual loss of substance, till the pulp is encroached upon, resulting in its destruction, and the loss of the organ itself,

The name "caries" would indicate a disease similar to that which attacks the bones, but this process is dissimilar in every respect except in the name.

There were for a long time only two hypotheses, entirely adverse to each other, explaining dental caries. The chemical and the vital theory. The chemical accounted for the disease by the action of acids; whilst the advocates of the vital theory, claimed dental caries to be a veritable malady, caused by an organic change in the tooth substance, and reaction against external irritants. The adherants of the chemical theory regard the teeth as nothing more than any other inanimate substance, to be acted upon by acids, without any inherent power whatever of reaction against deleterious agents.

There are others who differ entirely from the advocates of these extreme ideas, and assert the parasitic character of caries, attributing every step in the progress of decay to the action of animal or vegetable parasites.

Histologic investigators have detected great numbers of infusorial animalcules in the mucous covering the teeth and in carious cavities, which they say produce a kind of putrefaction, which after attacking the enamel cuticle, consumes the enamel and dentine in succession.

Another investigator, M. Klencke, adopts the putrefaction theoiy, but discovers several other species of caries; one commencing in the pulp cavity, that he denominates central caries, in contra-distinction to the common peripheric caries. He subdivides the latter into three different kinds,

"1. A soft caries caused by putrefaction.

2. A soft caries due to the proliferation of a vegetable parasite, called protococcus dentalis.

3. A dry caries, caused by the action of chemicals upon the dental tissues, and is independent of any parasitic influence."

Mr. Tomes is the first, I believe, to describe the histologic alterations in the tissues in which caries is the seat.

He takes the ground that these changes are caused, in the majority of cases, to use his own language, by an imperfect development, as well as a greater porosity of the tissues; a porosity which increases with the progress of the caries. To quote further, he says: "The canaliculi of the dentine present remarkable alterations during caries. In a cross-section we see them surrounded by a thickish sheath. One might say that the countour of the old cells of the dentine are re-established, and that the tissue is resolved into its primitive elements of formation."

According to Leber and Rottenstein the pathologic alteration proceeds along the canaliculi, toward the cavity of the pulp, giving, in most cases to the carious portion of the dentine the form of a brownish cone, with the base turned outwards. Around the cone is formed a zone, relatively transparent, in which the canaliculi contain dental fibrils, calcified, more or less long, and sometimes extending beyond the extremities of the canaliculi.

Mr. Tomes thinks that the irritation in the dentine and its organic reaction, superinduces this calcification of the canaliculi, He also concludes that because of this augmentation in the sensibility of the dentine that it is sensitive of itself, and not through its proximity to the pulp. We are surprised that such a man should come to such conclusion. This same author settles down to the conviction that acids are the principal cause of dental caries. The vitality of the parts are first destroyed, and then decomposed little by little. Rottenstein joins issue with him and asserts, that we can not produce artificially, by the action of acids upon the dentine, such histologic changes as we observe in carious teeth. Though this investigator admits that the decalcification, the softening, the destruction of the teeth are all phenomena due to the chemical nature of the process. But the dental tissues, while undergoing destruction, react against the action of the destroying agents, and this reaction is made manifest by the calcification of the dentinal fibrils in the parts which surround the carious, dentine, and also by the increased sensibility.

Mr. N. E. Newman, in his treatise, "*Sur la nature de la carie dentaire*," advances a most plausible theory, which accords with pathological conditions elsewhere in the human organism, to the effect, that the process of caries has an inflammatory nature, that injurious agents irritate the dentine, and produce phenomena of organic irritation, which end by causing the destruction of the tissues, just in the same manner that other organs and tissues are destroyed by active inflammation.

M. E. Magitot, in a treatise upon "Dental Caries," attempts to prove that the acids contained in the saliva, or mixed with it, are the cause of dental caries, and that the nature of this affection is purely chemical. He subjected human teeth to the action of various acids. They were destroyed, but the investigator found curious differences in the action of the various acids upon the dental tissues.

The teeth destroyed, however, were not subjected to microscopic examination, and consequently the test is defective and unsatisfactory.

M. Magitot does not attribute caries exclusively to chemical destruction. He admits the irritation of the pulp, which can arrest the destructive process by a calcareous exudation.

In this investigation of dental caries, some novel and unaccountable theories have been advanced.

We must accord to an Englishman, Mr. Bridgman, the credit of getting further off the track than any other investigator, according to my limited knowledge, in proclaiming that the teeth are not only formed by electricity, but may be destroyed by electrolysis. He arbitrarily attributes different electric properties to different parts of the teeth. The pulp vessels should be chared gwith negative electricity, the normal pulp with positive electricity, but, in a pathalogical state the surface of the dentine as well as the roots, should be charged with negative electricity. The proposition is too preposterous to admit of further notice.

From all the lights before us we may venture the assertion that there are but three theories that are entitled to respectful consideration, and they are chemical, vital and the parasitic.

The destructive process generally begins at the surface of the teeth or enamel. The dentine and cementum are rarely the seat of caries, before the enamel has been attacked.

Though there are some who advocate the central theory, or caries in the pulp cavity. Where this has occurred, we are disposed to believe, that the destructive agents had entered through some deep crevice, in the enamel and dentine, or disease, set up by a blow, or peculiar state of the system.

Caries of the enamel, then, constitutes the first stage of the process. The first pathalogical phenomena, however, make their appearance in the dentine even before the enamel is perforated.

The first thing to be seen, as an indication of decay, is a black or brownish point, in one of the furrows or folds of the crown. A section of the diseased portion will reveal a dark streak penetrating clear to the bottom of the furrow.

This dark spot increases with the disease, In these carious spots the enamel is changed into a chalky mass. Sometime it is softer than in its normal condition, but again it offers considerable resistance to the excavator, until we reach the deeper layers, which are more easily excavated.

This may be called the dry stage. When the enamel gives way and the dentine is attacked it is called humid caries.

This first impression made upon the enamel, or it may be the enamel cuticle, is undoubtedly the result of the action of acids. The peculiar color that accompanies decay seems to be consequent upon the change that has taken place in the organic structure of the enamel.

The enamel, being porous and less consistent, from the action of acids, fissures are made in it, which are at once filled by granulations of a vegetable fungus called leptothrix, which by the aid of acid liquids, persists in widening and deepening the breach.

In the dentine, even before it is reached by caries we may observe a brownish color more or less marked, and a lessening of consistency, due it is supposed, to a loss of a portion of the calcareous salts. And when the disease has progressed to a certain degree histologic changes take place which are attributed to the proliferation of the leptothrix, but were once regarded as the result of an active, vital action.

Caries ordinarily proceeds from the enamel to the dentine. Occasionally it takes its point of departure from the cement, or it may begin in the dentine, when denuded of its covering.

Its progress through the dentine is more rapid than in the enamel, which accounts for the cavity of decay being larger inside than externally. The minute canaliculi, so accessible to the liquids, offer a more extended surface to the action of destructive agents, and permit them to penetrate to the depths of the dentine, which is not possible with the enamel, it not having these canals.

The caries extends itself more rapidly in the direction of the canaliculi, than it does in the direction of the width of the tooth; the dentinal canaliculi as we said before, being more accessible to the destructive agents.

Enamel in its normal state is translucent, while dentine is of an opaque whitish or yellowish shade. The enamel loses its translucence by caries, while the dentine becomes more translucent and almost cartilaginous. The enamel loses its transparency by the action of acids. If we decalcify dentine by acids it becomes transparent. Acids produce the same effect out of the mouth as in it.

And though Mr. Tomes and M. Magitot endeavor to explain this transparency we find in carious dentine, otherwise than as the result of the action of acids, they fall short of the mark.

We will now notice some microscopic changes that take place in dentine, in a carious state.

There are two stages in which these changes may be observed, the preparatory period of decalcification and softening, and the period of direct decomposition. It has been thought by most investigators, that the dentine, deprived of its calcareous salts, is dissolved by a putrefactive process, but more recent critical investigation has revealed the fact that leptothrix performs an active part in the work of destruction. This fungus may be discovered in all the clefts and interstices of the decalcified tissue, which it reduces into small fragments It is thought equally probable that the same fungus, by its extension into the canaliculi and inter-globular spaces, plays an active and direct part in the absorption of the diseased tissues. This leptothrix is found in an astonishing quantity in the disorganized substance, which covers the superfices of a carious cavity. All the superficial layers are formed exclusively by the filaments of leptothrix, and by granular masses.

If we make sections of the diseased tooth, we will find that each layer as we progress into the dentine, which is of a brown color, is united by masses of leptothrix, diminishing, however, as we go deeper. Leptothrix does not confine itself to pre-existent cavities, but penetrates deeply into the dentine; and it has been proven, in our opinion, that the minute dentinal canaliculi are the channels through which it penetrates the dental tissues.

Observers have heretofore proven the presence of leptothrix in cavities of decay, but they did not suppose it exerted any influence in the process of decomposition, which it certainly does.

It has been demonstrated, beyond all cavil, that by the proliferation of the minute and innumerable spores of this fungus, they enter the canaliculi, expand or dilate them, and through them, penetrate even to the pulp cavity. If we make transverse sections of a diseased tooth, we may see this process exemplified in a striking manner. We shall also find all the chinks or fissures of the enamel full of leptothrix, as well as in the dilated canals, and the inter-globular spaces of the dentine.

The progress of caries of the enamel may be summed up, as follows: "By the action of an acid, the enamel becomes porous, at some point, or loses its normal consistence. At the same time there is seen to appear a brown color, in consequence of the change which has taken place in its organic structure. There is formed at the surface, a bed of leptothrix, which probably penetrates the dental cuticle, if it still exists, and destroys it, chinks and fissures are opened in the enamel, which has become less consistent. Acid liquids and granulations of leptothrix penetrate there, while minute fragments become detached, and are promptly enveloped by the elements of leptothrix, which joined to the continued action of the acids, hasten the dissolution of the tooth.

Caries of the dentine, as in the enamel, undergoes certain transformations, even before decay sets in, by assuming a brownish color, and by a lessening of consistency, due to the loss of a portion of calcareous salts. Caries of the dentine is much more rapid, than in the enamel, because of its structure; the accessibility of its canaliculi to liquids, and because a cavity being formed, the food and other destructive agents are confined in it, and are not so liable to be washed out, while cleansing the mouth. The main cause, however, lies in its canaliculous structure. The caries extends more rapidly in the direction of these canals, than in the width of the tooth.

If we divide a carious tooth lengthwise we may detect a brownish cone, with its base turned outward, even before any destruction of the dentine has taken place; and even while the enamel still has its polish, and has lost none of its substance.

In transverse sections, it is easy to see that the canaliculi are enlarged by the accumulation of a finely granular substance in their interior.

These histologic changes, once thought to be the result of an active vital action, are now attributed to the proliferation of leptothrix.

Leber and Rottenstein express the opinion that the brownish color of the cones, in the dentine, as in the enamel, is due to the decomposition of the organic portions of the tooth. The intensity of this color differing in the dentine, as in the enamel, in proportion to the density of the tooth, and the slow or rapid progress of the disease.

The canaliculi, by an accumulation in their interior of a finely granular substance, become gradually larger, till they reach a considerable size. We will not consume time, by going into detail, as to the changes that take place in the walls and inter-tubular spaces.

We will state that all the changes we have enumerated, take place in artificial teeth, made of hippopotamus ivory, or natural teeth of substitution, which fact completely annihilates the vital theory. We feel warranted in the assertion, then, upon a review of the whole subject, that the dilatation of the canaliculi is caused by the proliferation of the spores, infinitely minute and innumerable, of the fungus leptothrix, which, having a mobility of its own insinuates itself into the interior of the canals; and by its constant development, and the influence of the morbid action, and surrounding walls, the softened dentine becomes decomposed into irregular parcels, which separate, completing the destruction of the tissues.

But for the want of space, we might adduce the various theories as to the cause of the deposit of calcareous salts in the dentinal canals. The most plausible of which is that it is done by purely a chemical process. M. Magitot believes that these deposits result as an exudation from an irritated pulp; a kind of reparative process, as is sometimes found in the formation of new dentine in the pulp cavity, but his reasoning is, in our opinion, unsound. These calcareous deposits are more abundant when the caries is at a stand-still. or progressing slowly. We are not prepared to decide whether or not they retard or advance the march of caries. But we are satisfied that they are not the result of any vital or inflammatory action of the dentine, from the fact that the same thing occurs in teeth out of the mouth, when subjected to the proper tests. It has been supposed, by most dentists, that the tooth decays more rapidly after the death of the pulp. This may be true, to some extent, but not because the pulp exercises any conservatory action, excepting perhaps, in the new dentine which it throws out for its own temporary protection, but because the cavity, as I have before said, by the time the pulp is reached is in a condition to retain the destructive agents.

### CAUSES OF CARIES.

Upon a careful review of the opinions and experiments of our best investigators, I am prepared to assert that there are but two prominent phenomena, ever present and active in the process of dental caries, viz: the action of acids, and the development of a vegetable parasite, the leptothrix buccalis.

It is true there is a long list of predisposing causes which favor the attack of these injurious agents. The enamel itself, is subject to various anomalous conditions, congenital and otherwise, that renders it peculiarly liable to be assailed, as manifested in the honey-combed development, fissures from sudden changes, from hot to cold fluids, deficiency of calcareous salts in the enamel prisms; congenital patches, etc., certain specific constitutional diseases, such as syphilis, as well as maladies of the mouth, deranged state of the stomach; deficiency of secretion of saliva, as is often the case in sickness, thus preventing the neutralizing or diluting of the acids in the oral cavity, are some of the predisposing causes of caries.

We will notice some of the effects that acids produce upon the teeth. By actual experiments, it is demonstrated that it does not require strong acids to separate the phosphoric and carbonic acids from the lime contained in the tooth substances. Even water that contains carbonic acid will dissolve the calcareous salts. And it seems from a circumstance that transpired under the eye of Mr. Spence Bate, that water alone can dissolve the teeth. A lady having two sets of artificial, human teeth, she placed one set in water to preserve it till she had worn out the other. At the expiration of seven years, the set that she had kept in water was as much corroded as the one she had worn in the mouth. This case corroborates a statement made by Wedl and Heider, that at the end of ten days, fungi had attacked the enamel and dentine of teeth that had been kept in pure water. And that in a few weeks the tissues were pierced with holes like a sieve.

All mineral, as well as vegetable acids, act promptly on the teeth. "In forty-eight hours acetic, citric and malic acids will corrode the enamel so that you may scrape a great portion of it away with the finger nail."

Acid tartrate of lime, having a greater affinity for the lime of the tooth than for its own base, will rapidly destroy the enamel.

Grapes in forty-eight hours, will render the enamel of a chalky consistence. Vegetable substances are inert till fermentation takes place, and acetic acid is formed. Sugar has no deleterious effect, only in the state of acetous fermentation. Animal substances exert no injurious effect until putrefaction is far advanced.

The different acids as used in medicines, as shown in an article in *The "American Journal of Dental Science."* exert precisely similar effects upon the teeth as those just mentioned. M. Montegazza, instituted such tests as proved that sugar when changed by fermentation into lactic and acetic acid, as well as vinegar, strong or dilute, and lemon juice acted on the teeth.

M. Magitot, from his experiments, distinguishes "four categories of substances in relation to their action on the teeth."

1. Substances which attack alike all the tissues of the teeth. viz: sugar, only in state of fermentation, lactic, butyric, citric, malic, and carbonic acids, the products of the decomposition of albumen and albuminoid substances.

2. Substances which have the particular and exclusive property of destroying the enamel: alum, oxalic acid, and its acid salts.

3. Substances which act exclusively upon the dentine and upon the cement: acetic, tartaric acid and their salts, and tannin.

4. Substances which have no action upon the dental tissues, as common salt, and a great majority of other neutral substances which are found in the mouth.

Leber and Rottenstein, differ with M. Magitot as to the action of tartaric and acetic acid attacking the dentine, and not the enamel. They claim that both are alike affected.

Where there is such a contrariety of opinion as there is on this subject of dental caries, it is difficult, at times, to arrive at just conclusions.

Sugar has been charged as exerting an injurious action upon the teeth. And when we take into account the predominance of saccharine matter in our ordinary food, much less the deserts, fruits, candies and condiments that are brought into daily contact with the teeth, we must decide the charge a just one.

Malic, citric and tartaric acids are found in fruits Sour milk contains lactic acid. And where these various acids are taken directly into the mouth they form, as the result of decomposition.

The mucous membrane of the buccal cavity, when irritated, has been known to give out an acid secretion. The surface of the teeth and gums too, when irritated, from the abnormal state of the secretions, or from acid fermentation of particles of food, show an acid reaction.

M. Oehl has thrown considerable light upon the action of the buccal fluids upon elementary debris, or particles of food remaining in the mouth.

"He collected one portion of saliva after the ingestion of amylaceous substances, and another after the mouth had been thoroughly rinsed. The two liquids were filtered and left to themselves.

The former, which contained starch, reduced sulph-copper, proving that a portion of the starch had been converted into sugar. It remained several days in a state of acid fermentation before passing into the state of putrefaction, with alkaline reaction. In the second liquid, which contained only pure saliva, there was no acid reaction, but on adding starch or sugar to this saliva, primatively pure, in like manner as in the former, an acid fermentation was seen to take place."

He further more says, that, where sugar or starch was held in the mouth, when its reaction was alkaline, an acid reaction was formed at the end of from twenty to forty minutes, and even sooner when grape sugar was used.

It results then, when sugar or starch are mixed with the saliva, an acid fermentation takes place in the mouth, identical with that in the experiment. The secretions of the parotid and sub-maxillary glands have the power of converting starch into sugar.

We find, therefore, a continued source of acidity in the work of fermentation that takes place in the mouth.

The veritable nature of the acids found in the mouth, has not been ascertained by any direct experiment, however, it is generally conceded to be lactic acid.

Lactic acid, is produced as a general rule by the fermentation of sugared liquids, and others, when there are present at the same time albuminoid substances. In the buccal cavity, these very conditions are fulfilled. Albumen, is contained in the saliva, as well as carbonate of lime, in considerable quantity in the form of tartar.

It is thought to be exceedingly probable that leptothrix also plays a role in the acid fermentations of the mouth, and in other respects, constitutes one of the chief factors in dental caries.

As we remarked, in the beginning, the action of acids alone, does not produce the identical phenomena that we witness in caries of the teeth, though even very dilute acids dissolve the dental tissues. The difference between caries and the effect that acids produce is palpable.

Caries proceeds slowly in the enamel, while acids attack it vigorously, and rapidly change it into a chalky mass. Just the reverse effect is produced in the dentine. Acids penetrate it slowly, by gradually dissolving out its lime salts, while caries proceeds promptly along the canaliculi. This difference of progress, must undoubtedly be attributed to the dilatation of the canals by the elements of the fungus leptothrix, which glide readily into their interior, and favor the passage of the acids into the deep seated tissues.

The buccal cavity, is hardly ever free from leptothrix. Out of forty persons of different professions, and leading different kinds of lives, examined by Mr. Bowditch, he found in almost all of their mouths vegetable and animal parasites. There were none free from them but those who had used soap once a day, and had rinsed their mouths several times a day.

These parasites were not affected by the ordinary means employed for cleansing the teeth. While it appears that soap destroys them.

This fungus, though ordinarily found at the surface of the oral cavity, is seen to penetrate into the interior of the teeth during the progress of the disease. It is true that the enam l must have lost its density by the action of acids, to pave the way for the inroads of these parasites into the deeper tissues. It is scarcely possible for the fungi to disorganise the enamel in its normal condition; and were it not for the canals in the dentine, the resistance, in all probability, would be too great for them. But it is evident that after leptothrix has once gained a foothold upon these tissues, it produces, by its extension, especially in the dentine, effects of softening and disorganization much more rapidly than can be accomplished by acids alone.

We decide, then, that in the dry stage of caries, the work of destruction is due entirely to acids, but so soon as the dentine is encroached upon the leptothrix, begins a role more constant and more active than is exerted by acids.

One peculiarity worthy of remark is, that the development of the fungus, seems to be favored by a neutral or slightly acid medium, while a strongly alkaline liquid is destructive to it. Dr. Bowditch, has observed that the parasites entirely disappear when the mouth is rinsed with a solution of soap; and M. A. Vogel, has found that though other fungi would develop in pure water, or in solutions of salts without alkaline reaction, and especially in solutions of sugar, no proliferation could take place in slightly alkaline solutions.

In view then of all the foregoing considerations, I enquire what is our duty as dentists in the premises. Shall we allow our patrons or the general public, even to continue to be blinded as to the causes that operate in the destruction of their teeth? Shall we, as a profession, turn our back upon suffering humanity, because for sooth our calling might prove less remunerative, were the people better informed as to the causes, and provided with prophylactics against the decay of their teeth?

We can scarcely hope for that advance in science, that will point out the cause and provide a remedy for every disease to which flesh has fallen heir. Still the paramount duty of the dentist and the physician is to ameliorate, if not arrest the progress of disease.

Dentistry, has done more already than all other professions combined to improve the teeth of succeeding generations, by the agitation of the subject, and arousing the people to the importance of bone and tooth making food. It has been but a few years past that you could procure no other but demineralized food at hotels or at our grocers; while at present, oat meal, cracked wheat, rye flour, and graham bread are staple articles on sale everywhere; made such by the demand for them, under an enlightened consciousness of their utility. Whenever investigators shall have solved this vexed question, the cause of dental caries, should not mankind reap the benefit of such discovery?

I am proud to say that there are many noble philanthropists in our profession, who have manifested their solicitude for the prevention of dental diseases by their efforts to enlighten the masses on the subject of tooth making food, and dental hygiene.

Let every one of us consider it our imperative duty not only to combat disease by repairing its ravages, but to prevent it.

Our profession may accomplish wonders in the development and improvement of the physique, as well as the teeth of the generations that succeed us.

We should educate the people to the importance of a proper diet for the nourishment of the teeth of their children, and impress upon them not only the importance of a radical change in the use of bread stuffs, but the importance of the use of the brush and cleanliness of the oral cavity. And last, but not least, if soap will destroy vegetable and animal parasites, let us popularize the daily use of soap or saponaceous compounds.

Feeling a just pride then, in our calling, and a determination to spread such truths of dental science over our land, as must result in good, let us take comfort from the lines of Longfellow:

"Lives of great men, all remind us
We can make our lives sublime,
And departing, leave behind us,
Foot prints on the sands of time.
Let us then be up and doing
With a heart for any fate,
Still achieving, still pursuing,
Learn to labor and to wait."

